# Development and validation of a novel clinical prediction model to predict the 90-day functional outcome of spontaneous intracerebral hemorrhage

**DOI:** 10.3389/fneur.2023.1260104

**Published:** 2023-09-26

**Authors:** Zhi Geng, Tao Guo, Ziwei Cao, Xiaolu He, Jing Chen, Hong Yue, Aimei Wu, Lichao Wei

**Affiliations:** ^1^Department of Neurology, First Affiliated Hospital, Anhui Medical University, Hefei, Anhui, China; ^2^Anhui Province Key Laboratory of Cognition and Neuropsychiatric Disorders, Hefei, Anhui, China; ^3^Collaborative Innovation Centre of Neuropsychiatric Disorder and Mental Health, Hefei, China; ^4^Center for Biomedical Imaging, University of Science and Technology of China, Hefei, China; ^5^Department of Neurology, The Second People's Hospital of Hefei, Hefei, Anhui, China; ^6^Department of Neurosurgery, Huashan Hospital, Fudan University, Shanghai, China; ^7^Neurosurgical Institute of Fudan University, Shanghai, China; ^8^Shanghai Clinical Medical Center of Neurosurgery, Shanghai, China; ^9^Shanghai Key Laboratory of Brain Function and Regeneration, Huashan Hospital, Fudan University, Shanghai, China

**Keywords:** spontaneous intracerebral hemorrhage, prognosis, prediction model, nomogram, national institute of health stroke Scale (NIHSS)

## Abstract

**Background:**

Spontaneous intracerebral hemorrhage (SICH) is associated with high mortality and disability. Accurately predicting adverse prognostic risks of SICH is helpful in developing risk stratification and precision medicine strategies for this phenomenon.

**Methods:**

We analyzed 413 patients with SICH admitted to Hefei Second People's Hospital as a training cohort, considering 74 patients from the First Affiliated Hospital of Anhui Medical University for external validation. Univariate and multivariate logistic regression analyses were used to select risk factors for 90-day functional outcomes, and a nomogram was developed to predict their incidence in patients. Discrimination, fitting performance, and clinical utility of the resulting nomogram were evaluated through receiver operating characteristic (ROC) curves, accuracy, sensitivity, specificity, positive predictive value (PPV), negative predictive value (NPV), calibration plots, and decision curves analysis (DCA), respectively.

**Results:**

Of the 413 patients, 180 had a poor prognosis. Univariate analysis showed significant variance of age, systolic pressure, intraventricular hemorrhage (IVH), Glasgow Coma Scale (GCS) scores, National Institute of Health Stroke Scale (NIHSS) scores, and hematoma volume between the groups (*p* < 0.05). Logistic multivariate regression analysis showed that age, IVH, NIHSS, and hematoma volume were associated with unfavorable outcomes. Based on the results, a nomogram model was developed with an area under the ROC curve of 0.91 (95% CI; 0.88–0.94) and 0.89 (95% CI; 0.80–0.95) in the training and validation sets, respectively. In the validation set, the accuracy, sensitivity, specificity, PPV, and NPV of the model were 0.851, 0.923, 0.812, 0.727, and 0.951, respectively. The calibration plot demonstrates the goodness of fit between the nomogram predictions and actual observations. Finally, DCA indicated significant clinical adaptability.

**Conclusion:**

We developed and validated a short-term prognostic nomogram model for patients with SICH including NIHSS scores, age, hematoma volume, and IVH. This model has valuable potential in predicting the prognosis of patients with SICH.

## 1. Introduction

SICH refers to the occurrence of a blood clot in the brain parenchyma without any associated trauma or surgical intervention (1). SICH is a type of stroke with high mortality and disability rates that causes significant stress to individuals and society (2). The overall incidence of stroke has declined in recent decades; however, the incidence of SICH remains controversial. Some studies have demonstrated a decrease in SICH incidence in individuals aged <75 years (3) or a decline in hypertension-related SICH (4). The incidence of stroke is relatively stable in high-income countries; however, it has been increasing in low-income countries (5). The incidence of SICH is expected to increase with the aging of the global population.

Although the incidence of SICH is lower than that of ischemic stroke, the associated death and disability rates are higher, causing difficulties for individuals and families (6). Extensive studies on risk factors for SICH, as well as preliminary studies on its prognosis, have been conducted. The prediction of disease prognosis is an important part of clinical research; therefore, researching prognostic factors for SICH is important, especially in determining controllable risk factors. With the emergence of artificial intelligence (AI), radiological techniques have become extensively utilized for predicting prognosis in ICH patients. However, it is important to note that this method is still in the research phase, and its predictive performance needs to be further validated (7, 8).

The nomogram is a visual prediction tool that assigns different scores to different states of each variable to obtain the probability of an ending. Currently, there are several prediction models to predict patients with SICH; however, they may be controversial owing to their predictive power and clinical application ability (9, 10). It is necessary to optimize the predictive capability of the model and improve its patients with SICH.

## 2. Materials and methods

### 2.1. Patients

Patients with SICH admitted to the Department of Neurology at the Hefei Second People's Hospital between November 2018 and February 2022 were enrolled in this study. Patients meeting the following criteria were included: (1) those who signed informed consent, (2) age ≥18 years, and (3) SICH diagnosis within 7 days of symptom onset. The exclusion criteria were as follows: (1) hemorrhage due to trauma, intracranial vascular malformation, neoplasm, or any other presumed cause of secondary SICH; (2) isolated IVH; (3) any type of surgical hematoma evacuation (including external ventricular drainage); and (4) loss to follow-up. Poor and good 90-day functional outcomes were defined as modified Rankin scale (mRS) scores of 3–6 and 0–2, respectively (11, 12). All patients have undergone conventional treatment for SICH in accordance with the guidelines (13).

**Figure 1:**
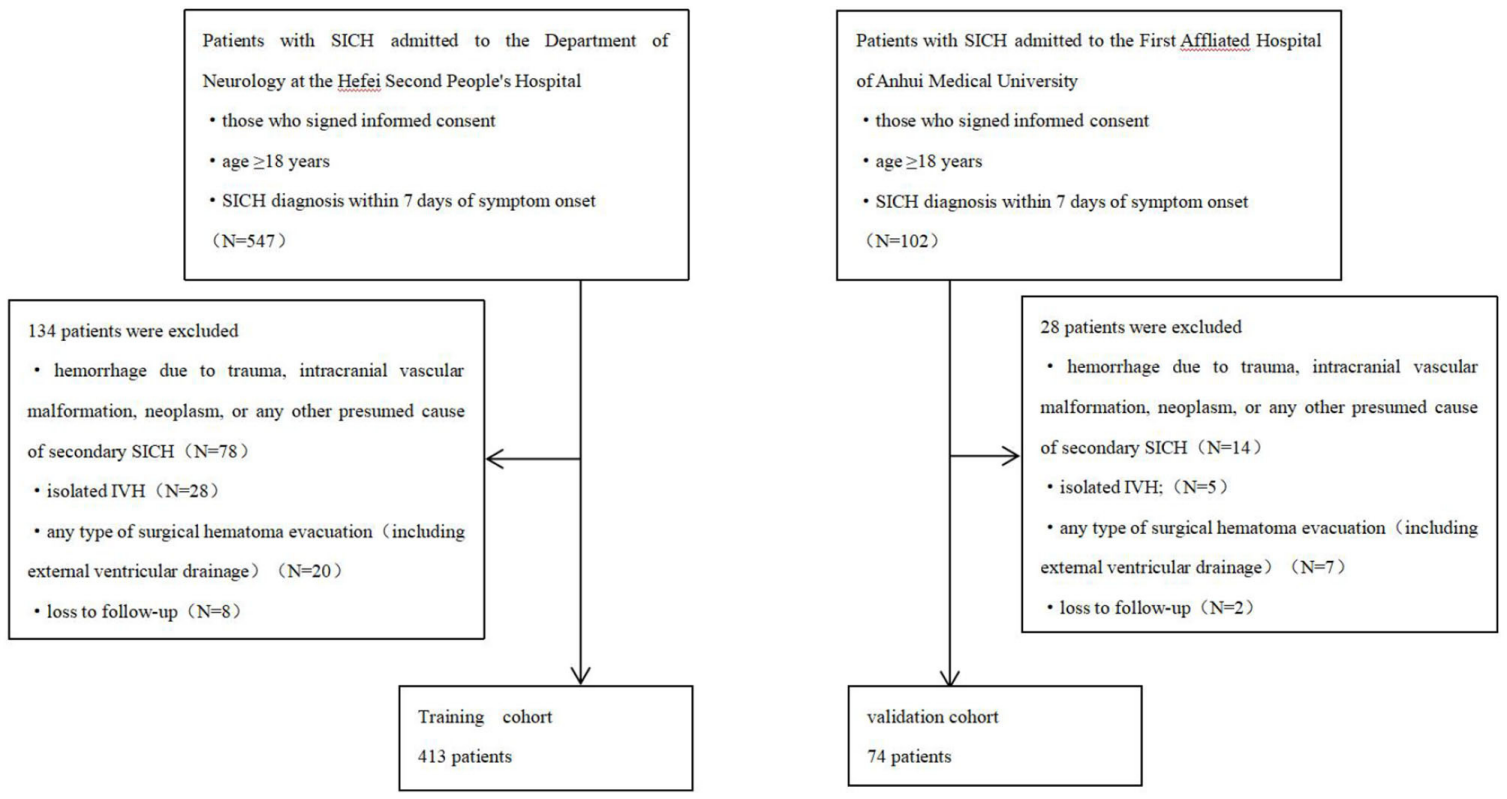
Selection flowchart of included and excluded patients.

An independent cohort of patients was enrolled at the First Affiliated Hospital of Anhui Medical University between January and June 2022. Patients were screened using the same inclusion and exclusion criteria to serve as an external validation cohort (Figure 1).

### 2.2. Data acquisition

Demographic and clinicopathological variables of the enrolled patients were collected from their medical records and questionnaires. Data collected included gender, age, systolic blood pressure, diastolic blood pressure, NIHSS scores, GCS scores, hematoma volume, hemorrhage location, intraventricular hemorrhage, smoking, drinking, and basic diseases, including hypertension and diabetes. The measurements in this study, including GCS, NIHSS, hematoma volume, and MRS, were all conducted by neurologists (more than 10 years of clinical experience in neurology).

### 2.3. Statistical methods

All data analysis and drawings were completed using the Empower(R) (www.empowerstats.com, accessed on June 1, 2022, XandY Solutions, Inc Boston, MA, USA) and R 3.6.3 (http://www.R-project.org). Count data were expressed as cases and rates using the chi-square test or Fisher's exact probability method; measures that conformed to a normal distribution were expressed as (mean ± standard deviation) using the two-sample *t*-test; measures that did not conform to a normal distribution were expressed as median (interquartile range) using the Mann–Whitney *U*-test. We explored the relationship between all variables and poor outcomes by the univariate analysis, including variables with a *p-*value of < 0.05 that were included in the multivariate logistic regression analysis to identify independent risk factors for the poor outcomes. To avoid multicollinearity between variables, we evaluated the variance inflation factor (VIF) between all variables, and variables with VIF >5 were excluded from the final analysis. Statistical significance was set at a *p-*value of < 0.05 (two-tailed).

Based on the independent risk factors for the poor outcomes, we constructed a nomogram for the training set. ROC curves were separately used to evaluate the accuracy of the nomogram model in the training and external validation sets. We plotted the calibration curves to demonstrate the association between the actual probability and predicted probabilities, and the clinical unity was evaluated by DCA.

## 3. Results

### 3.1. Clinical characteristics

Table 1 shows the demographic information and related risk factors, which were comparable between the training and validation cohorts. The training cohort comprised 145 women (34.11%) and 268 men (64.89%). In total, 233 (56.42%) patients had good outcomes, and 180 (43.58%) patients had poor outcomes. The validation cohort included 20 women (27.03%) and 54 men (72.97%). In total, 48 (64.86%) patients had good outcomes, and 26 (35.14%) patients had poor outcomes. Table 2 shows the results of the univariate and multivariate regression analysis for the various variables. The factors that varied statistically in the univariate analysis included age (*p* = 0.0167), NIHSS scores (*p* < 0.0001), GCS scores (*p* < 0.0001), hematoma volume (*p* < 0.0001), systolic pressure (*p* = 0.0006), and IVH (*p* < 0.0001). The other variables showed no significant differences. Outcome quality was used as a binary classification-dependent variable. Furthermore, the multivariate regression analysis showed that the age (OR = 2.38, 95% CI, 1.33–4.28, *p* = 0.0036), hematoma volume (20–40 ml, OR = 4.38, 95% CI, 1.84–10.44, *p* = 0.0009; ≥ 40 ml, OR = 8.01, 95% CI, 1.48–43.40, *p* =0.0158), NIHSS scores (OR = 1.34, 95% CI, 1.23–1.45, *p* < 0.001), and intraventricular hemorrhage (OR = 2.21, 95% CI, 1.05–4.64, *p* = 0.0371) were independent risk factors for the development of poor outcomes.

**Table 1:** Demographics and clinical characteristics of study in the training and validation cohorts.

**Group**	**All data**	**Train data**	**Test data**	***P*-value**
N	487	413	74	
SBP (mmHg)	162.71 ± 26.85	163.69 ± 27.29	157.23 ± 23.68	0.057
DBP (mmHg)	93.31 ± 17.30	93.90 ± 17.30	89.99 ± 17.04	0.073
GCS scores	12.99 ± 3.38	12.86 ± 3.42	13.70 ± 3.08	0.047
NIHSS scores	9.70 ± 9.59	10.08 ± 9.73	7.59 ± 8.56	0.040
Age (years)				0.460
< 65	322 (66.12%)	209 (50.61%)	34 (45.95%)	
≥ 65	165 (33.88%)	204 (49.39%)	40 (54.05%)	
Sex				0.176
Male	322 (66.12%)	268 (64.89%)	54 (72.97%)	
Female	165 (33.88%)	145 (35.11%)	20 (27.03%)	
Hypertension				< 0.001
No	119 (24.44%)	113 (27.36%)	6 (8.11%)	
Yes	368 (75.56%)	300 (72.64%)	68 (91.89%)	
Diabetes				0.942
No	429 (88.09%)	364 (88.14%)	65 (87.84%)	
Yes	58 (11.91%)	49 (11.86%)	9 (12.16%)	
Smoking				0.071
No	375 (77.00%)	312 (75.54%)	63 (85.14%)	
Yes	112 (23.00%)	101 (24.46%)	11 (14.86%)	
Drinking				0.007
No	359 (73.72%)	295 (71.43%)	64 (86.49%)	
Yes	128 (26.28%)	118 (28.57%)	10 (13.51%)	
Hematoma volume (ml)				0.160
< 20	383 (78.64%)	322 (77.97%)	61 (82.43%)	
20–40	55 (11.29%)	45 (10.90%)	10 (13.51%)	
≥ 40	49 (10.06%)	46 (11.14%)	3 (4.05%)	
IVH				0.559
No	355 (72.90%)	299 (72.40%)	56 (75.68%)	
Yes	132 (27.10%)	114 (27.60%)	18 (24.32%)	
Infratentorial hemorrhage				< 0.001
No	431 (88.50%)	375 (90.80%)	56 (75.68%)	
Yes	56 (11.50%)	38 (9.20%)	18 (24.32%)	
Hemorrhage location				0.869
Deep	402 (82.55%)	340 (82.32%)	62 (83.78%)	
Lobar	85 (17.45%)	73 (17.68%)	12 (16.22%)	
Outcome				0.176
Good outcome	281 (57.70%)	233 (56.42%)	48 (64.86%)	
Poor outcome	206 (42.30%)	180 (43.58%)	26 (35.14%)	

SDP, systolic blood pressure; DBP, diastolic blood pressure; GCS, Glasgow Coma Scale; NIHSS, National Institute of Health stroke scale; IVH, intraventricular hemorrhage.

**Table 2:** Univariate and multivariate logistic analyses of the training cohort.

	**Univariable logistic regression**	**Multivariable logistic regression**
**Age (years)**	**OR (95% CI)** ***P*****-value**	**OR (95% CI)** ***P*****-value**
< 65	1.0	1.0
≥ 65	1.61 (1.09, 2.39) 0.0167	2.38 (1.33, 4.28) 0.0036
**Sex**		
Male	1.0	
Female	1.28 (0.86, 1.93) 0.2280	
**Hypertension**		
No	1.0	
Yes	0.96 (0.62, 1.49) 0.8673	
**Diabetes**		
No	1.0	
Yes	0.72 (0.39, 1.34) 0.3046	
**Smoking**		
No	1.0	
Yes	0.68 (0.43, 1.08) 0.1061	
**Drinking**		
No	1.0	
Yes	0.77 (0.50, 1.19) 0.2336	
SBP (mmHg)	1.01 (1.01, 1.02) 0.0006	1.00 (0.98, 1.01) 0.4296
DBP (mmHg)	1.01 (1.00, 1.02) 0.1000	
**Hematoma volume**		
< 20	1.0	1.0
20-40	4.58 (2.34, 8.97) < 0.0001	4.38 (1.84, 10.44) 0.0009
≥ 40	45.47 (10.82, 191.12) < 0.0001	8.01 (1.48, 43.40) 0.0158
**Hemorrhage location**		
Deep	1.0	
Lobar	1.299(0.774, 2.181) 0.322	
**IVH**		
No	1.0	1.0
Yes	7.62 (4.61, 12.58) < 0.0001	2.21 (1.05, 4.64) 0.0371
**Infratentorial hemorrhage**		
No	1.0	
Yes	0.73 (0.37, 1.46) 0.3806	
NIHSS scores	1.31 (1.24, 1.38) < 0.0001	1.34 (1.23, 1.45) < 0.0001
GCS scores	0.56 (0.49, 0.65) < 0.0001	1.20 (0.96, 1.49) 0.1087

SDP, systolic blood pressure; DBP, diastolic blood pressure; GCS, Glasgow Coma Scale; NIHSS, National Institute of Health stroke scale; IVH, intraventricular hemorrhage.

### 3.2. Nomogram development

**Figure 2:**
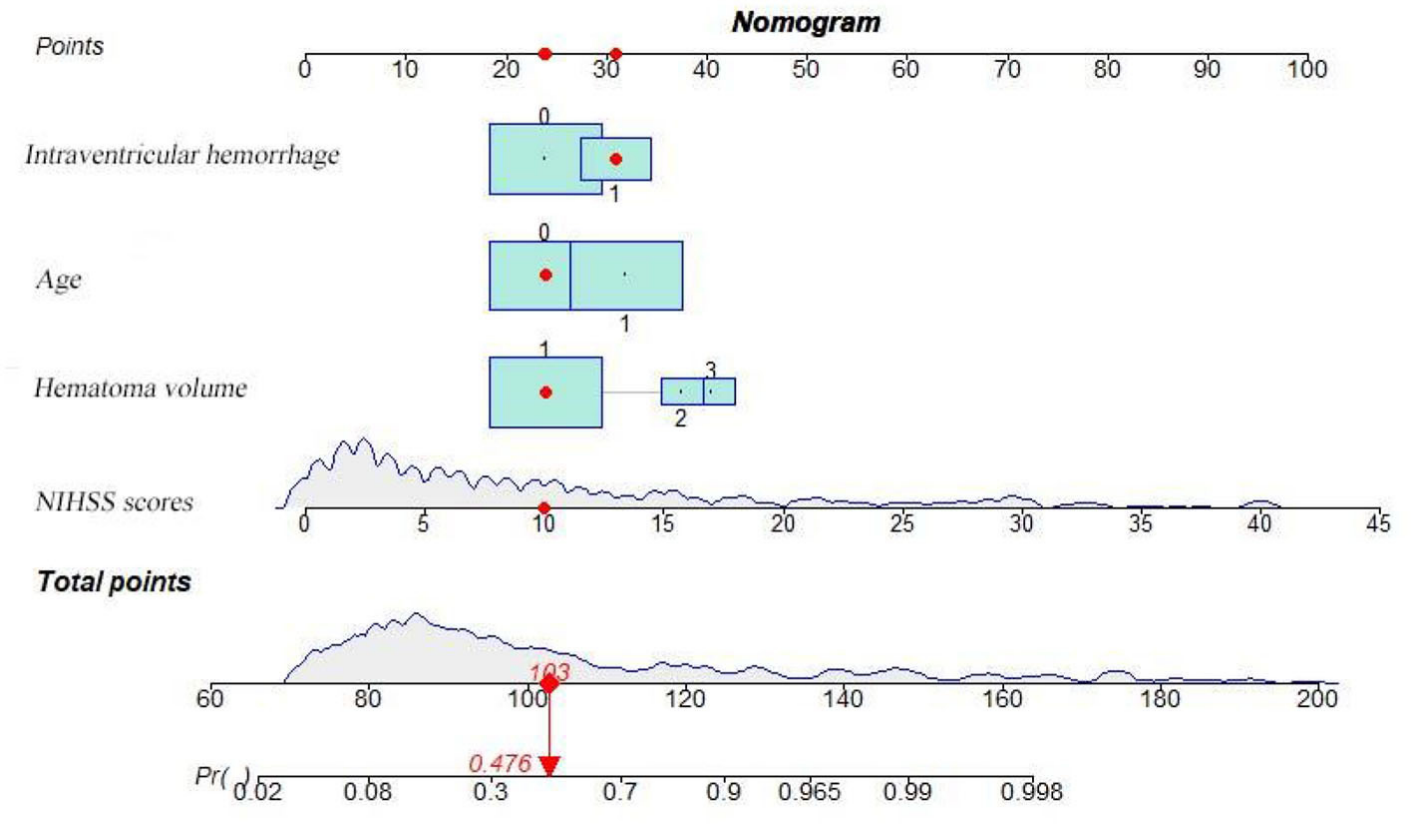
Nomogram for predicting the 90-day functional outcome of SICH. For intraventricular hemorrhage, 0 = no, 1 = yes. For age, 0 = (< 65 years), 1 = (≥ 65 years). For hematoma volume, 1 = (< 20 ml), 2 = (20–40 ml), 3 = (≥ 40 ml).

**Figure 3:**
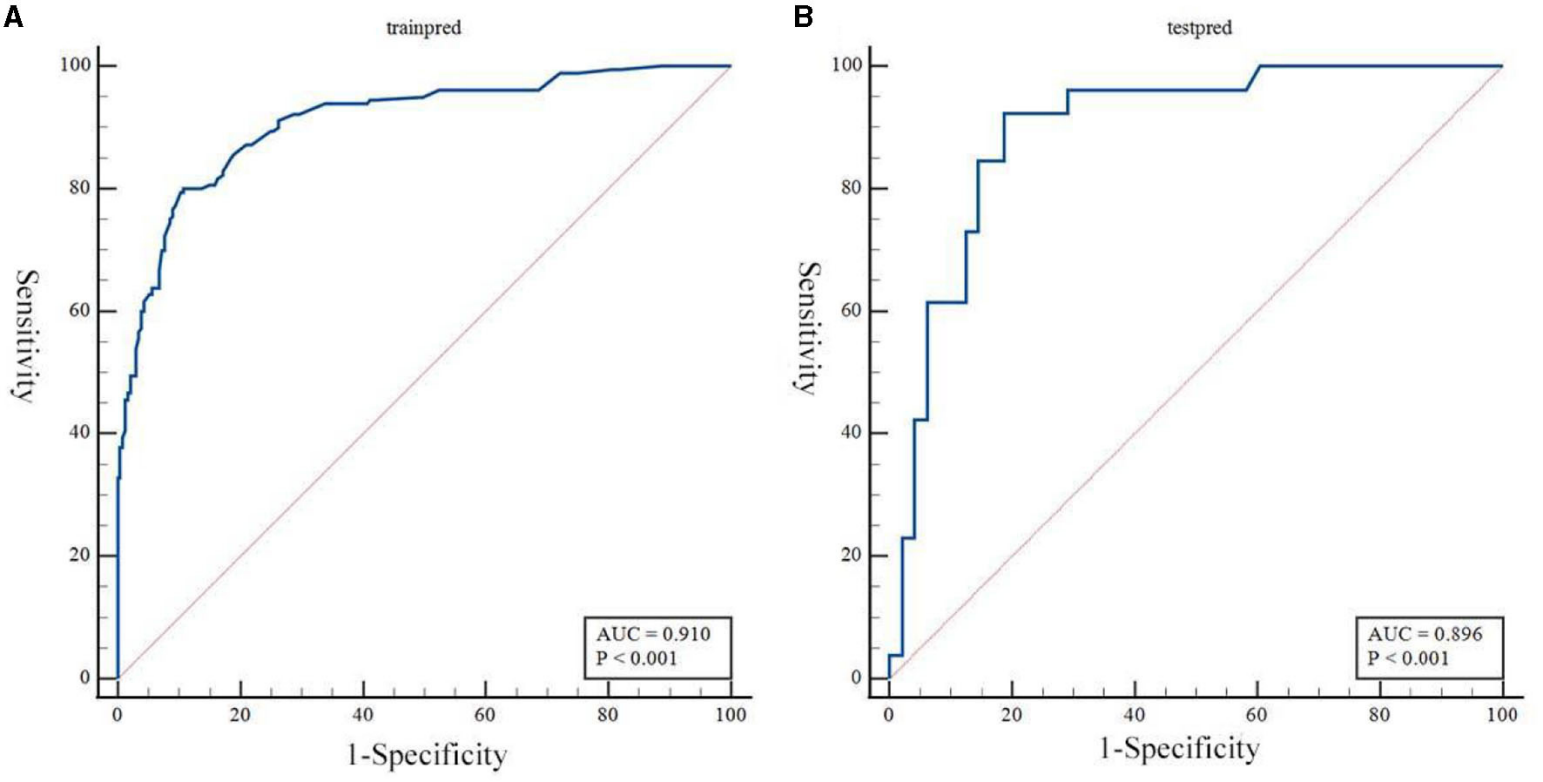
**(A, B)** Receiver operating characteristic curves to evaluate the discriminating capability of the nomogram in the training cohort and validation cohort.

**Figure 4:**
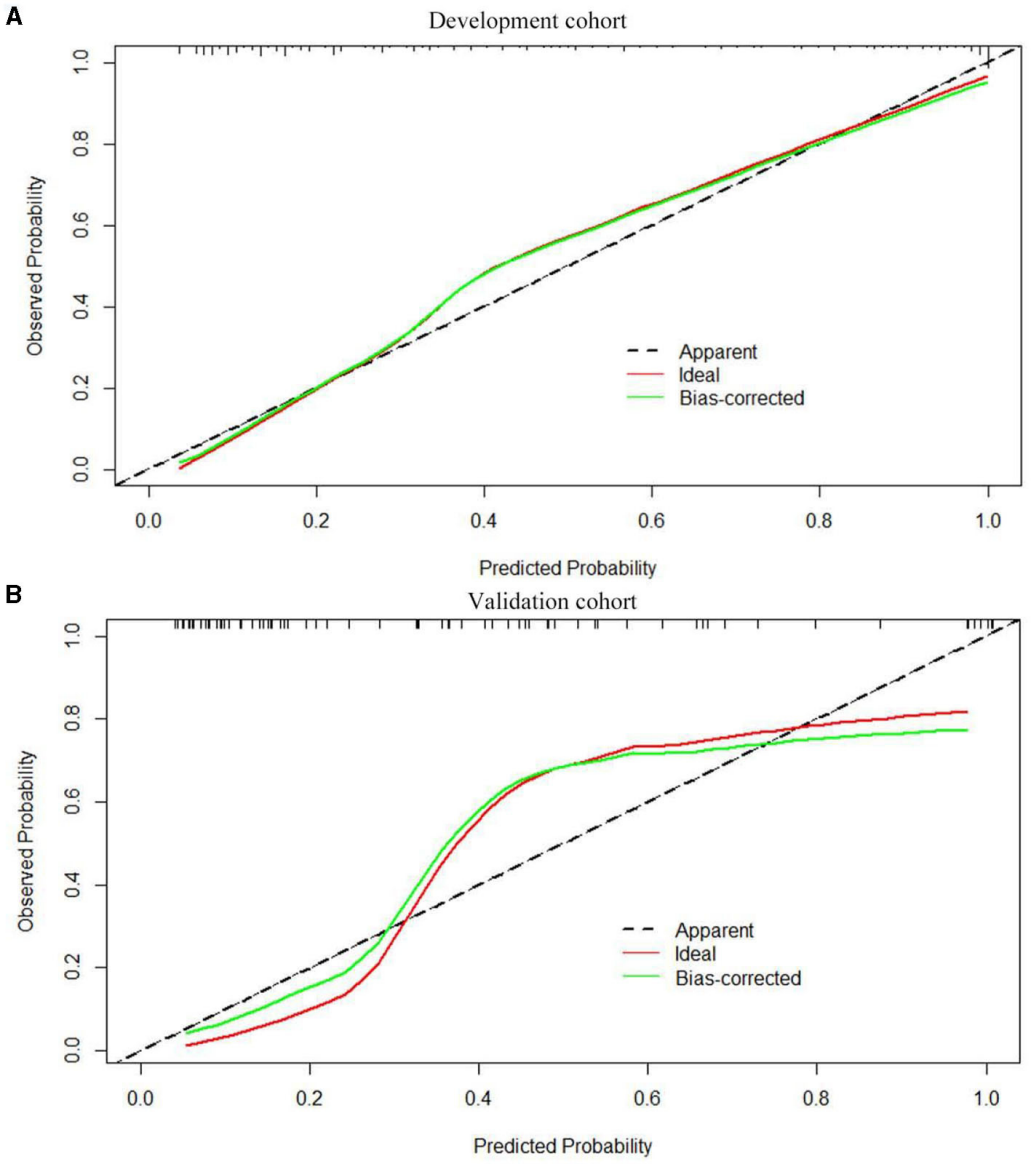
**(A, B)** The calibration curves for the nomogram in the training and validation cohorts.

**Figure 5:**
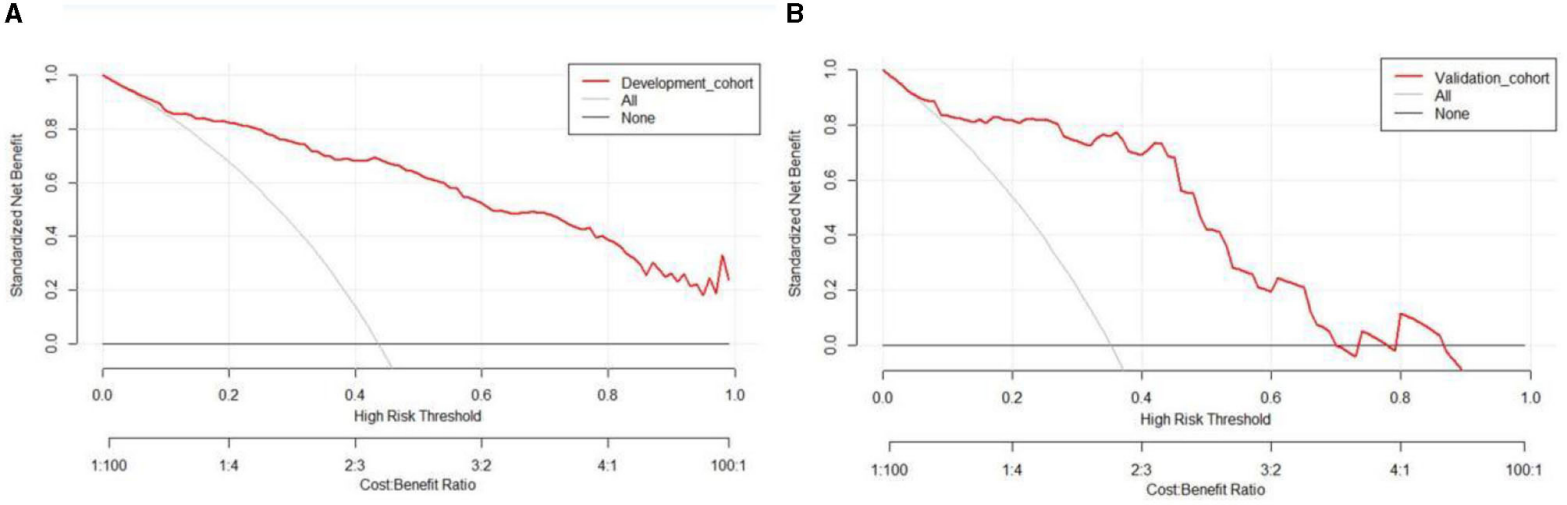
**(A, B)** Decision curves for the clinical nomogram in the development and validation cohorts.

Based on the results obtained, we developed a predictive model and generated a nomogram predicting the incidence of poor outcomes in patients with SICH (Figure 2). The prediction model demonstrated a good discriminatory ability, with an AUC of 0.91 (95% CI, 0.88–0.94; Figure 3A). The accuracy, sensitivity, specificity, PPV, and NPV of the model were 0.852 (95% CI, 0.852–0.853), 0.800 (95% CI, 0.742–0.858), 0.893 (95% CI, 0.853–0.932), 0.852 (95% CI, 0.799–0.906), and 0.852 (95% CI, 0.808–0.94), respectively. In addition, a calibration curve was generated by plotting the actual poor outcome (y-axis) against the predicted incidence risk (x-axis). The degree of fit between predictions and observations was good and was basically coincident, indicating that the prediction results were accurate (Figure 4A). In DCA diagrams, the nomogram showed a good clinical utility in predicting poor outcomes in patients with SICH (Figure 5A).

### 3.3. Validation of the nomogram

The nomogram was tested using the external validation method with an AUC of 0.89 (95% CI, 0.80–0.95; Figure 3B). The accuracy, sensitivity, specificity, PPV, and NPV of the model were 0.851 (95% CI, 0.848–0.855), 0.923 (95% CI, 0.821–0.999), 0.812 (95% CI, 0.702–0.923), 0.727 (95% CI, 0.575–0.879), and 0.951 (95% CI, 0.885–0.999), respectively. The calibration curve of the validation cohort was close to the ideal diagonal line (Figure 4B). The DCA diagrams showed a significant potential of the nomogram for clinical decision-making (Figure 5B).

## 4. Discussion

In this study, multiple regression analysis showed that NIHSS score, age, hematoma volume, and IVH were risk factors for poor short-term prognosis in patients with SICH, and a nomogram prediction model was constructed based on these clinical characteristics. The model has a good prognostic prediction ability and high clinical applicability and can therefore provide a reference value to aid in developing risk stratification and precision medicine strategies for patients with cerebral hemorrhage.

The NIHSS is an internationally recognized, comprehensive, and effective assessment tool for assessing neurological function in patients with SICH. The univariate analysis had both GCS and NIHSS scores as risk factors; however, only NIHSS scores were an independent risk factor for poor prognosis. Existing prognostic scales for SICH mostly use the GCS scores due to their simplicity and effectiveness. Some studies found that the ability to predict death after replacing the GCS scores with the NIHSS scores was weaker than the original ICH score, but the ability to predict neurological outcomes was enhanced (14). This may be related to the wider range of NIHSS scores because in addition to assessing patients' level of consciousness, the NIHSS also assesses neurological deficits (15). However, both the GCS and NIHSS are subjective indexes, and this may cause evaluation bias; therefore, this score should be affirmed with further evaluation. Therefore, an accurate and objective evaluation of a patient's onset or admission can help guide follow-up treatment.

Age is a risk factor for cerebrovascular disease, and SICH incidence increases with age (6). Advanced age was strongly associated with initial SICH severity as well as 90-day mortality or morbidity (16). As the patient's age increases, immunity decreases, reducing disease tolerance. This leads to more severe neurological damage and subsequently to an increased likelihood of other complications. Elderly patients often find it inconvenient to move due to their families' economic struggles and their own physical limitations. In addition, the medical policy lacks targeted treatment for elderly patients, leading to a poor prognosis. A retrospective study by Bernardo et al. identified increased age as an independent risk factor for high long-term mortality after SICH (17); however, some studies have suggested that age is not associated with SICH prognostic (18). Therefore, this topic remains controversial. This study also suggests that age is an independent risk factor for poor prognosis in patients with SICH at 90 days.

The amount of bleeding after a cerebral hemorrhage is an important indicator of its severity. Hematoma size has been reported to be closely related to prognosis in patients with SICH (19–21). In these patients, intracranial hematoma is involved in secondary brain injury as well as mechanical injury. Furthermore, hematoma size is closely related to intracranial pressure, with intracranial hypertension being an important factor in poor prognosis (22). The size of the hematoma on admission is significantly associated with short-term mortality (23). This corresponds to the results of the present study, which indicated that hematoma size on admission was an independent risk factor for poor 90-day prognosis in patients with SICH. Therefore, a large volume of intracranial hematoma after SICH can cause severe neurological deficits and secondary brain damage, resulting in poor prognosis. No clear criteria have been established for determining the standard hematoma volume in relation to SICH prognosis.

IVH occurs in 30–45% of patients with SICH (19). Its occurrence depends not only on the location and volume of the hemorrhage, as well as blood pressure fluctuations and abnormalities in the coagulation cascade (24, 25). The occurrence of new IVH, even of only 1 ml, appears to be a strong predictor of poor prognosis in patients with SICH (26). A follow-up analysis of the ATACH II trial by Okazaki et al. identified IVH as a risk factor for late 90-day neurological deterioration in these patients (27). Similar to the results of this study, SICH with IVH was identified as an independent risk factor for poor prognosis. However, a prospective study by Roeder et al. concluded that minor IVH (not causing obstructive hydrocephalus) was not associated with adverse outcomes or death after SICH (28).

There are still some limitations in this study. First, the relatively small sample size has limited persuasive power, and further studies should expand the sample size and increase the follow-up time. Second, a single external validation center was used in this study; therefore, further validation of the generalizability of this prediction model is required. In future studies, we will combine different regions and multiple external validation centers to optimize and validate the model. Third, this study did not combine multiomics, such as imaging and metabolomics, for model construction; however, its clinical applicability was increased since the predictive model was based on clinical characteristics. Fourth, the patients included in this study were non-surgical patients, so the results are not applicable to patients who underwent surgical treatment. In the following study, we will further expand the research population and establish a more complete prediction model.

## 5. Conclusion

In summary, NIHSS scores, age, hematoma volume, and IVH were found to be independent risk factors for the 90-day poor functional outcomes of SICH. In addition, the ROC curve, calibration plot, and DCA curve showed good predictive performance and calibration. Therefore, this model can help predict the 90-day functional outcomes of SICH.

## Data availability statement

The raw data supporting the conclusions of this article will be made available by the authors, without undue reservation.

## Ethics statement

The studies involving human participants were reviewed and approved by the Ethics Committee of Hefei Second People's Hospital and First Affiliated Hospital of Anhui Medical University. Written informed consent to participate in this study was provided by the patient/participants' OR patient/participants legal guardian/next of kin.

## Author contributions

ZG: Formal analysis, Writing—review and editing, Data curation. TG: Writing—original draft, Software, Supervision. ZC: Investigation, Methodology, Writing—original draft. XH: Formal analysis, Writing—review and editing. JC: Writing—original draft, Resources. HY: Writing—original draft. AW: Writing—original draft, Writing—review and editing, Data curation. LW: Writing—original draft, Writing—review and editing, Formal analysis, Investigation, Visualization.
